# Risk of Criminal Justice System Interactions in Young Adults with Attention-Deficit/Hyperactivity Disorder: Findings From a National Birth Cohort

**DOI:** 10.1177/10870547231177469

**Published:** 2023-05-30

**Authors:** Francesca Anns, Stephanie D’Souza, Conrad MacCormick, Brigit Mirfin-Veitch, Betony Clasby, Nathan Hughes, Warren Forster, Eden Tuisaula, Nicholas Bowden

**Affiliations:** 1 University of Auckland, New Zealand; 2 A Better Start National Science Challenge, New Zealand; 3 Nicholson Consulting, Wellington, New Zealand; 4 Donald Beasley Institute, Dunedin, Otago, New Zealand; 5 University of Sheffield, UK; 6 University of Otago, Dunedin, New Zealand; 7 Simply Resolution Limited, Wellington, New Zealand

**Keywords:** ADHD, criminal justice system interactions, neurodevelopment, integrated data infrastructure

## Abstract

**Objective:**

To examine criminal justice system (CJS) interactions and pathways through the justice system for young adults with ADHD compared to young adults without ADHD.

**Method:**

Nationwide 3-year birth cohort study using linked health and CJS data. Cox proportional hazards models were employed to examine associations between ADHD and police proceedings, court charges, court convictions, and incarcerations.

**Results:**

Young adults with ADHD were significantly more likely to interact with the CJS including police proceedings (hazard ratio [HR], 2.1 95% CI [2.0, 2.2]) court charges (HR, 2.2 95% CI [2.1, 2.3]), court convictions (HR, 2.3 95% CI [2.2, 2.4]), and incarceration (HR, 4.8 95% CI [4.3, 5.4]).

**Conclusions:**

Young adults with ADHD are overrepresented at all stages of the CJS. Results highlight the importance of early identification and responsivity to ADHD within the CJS and suggest that the NZ justice system may require changes to both areas to ensure that young individuals with ADHD receive equitable access to, and treatment within, the CJS.

## Introduction

Attention-Deficit/Hyperactivity Disorder (ADHD) is clinically defined as a neurodevelopmental condition characterized by the presence of pervasive and developmentally excessive levels of hyperactivity, impulsivity, and difficulties in attention (Mehta et al., 2019; Sayal et al., 2018; Young & Cocallis, 2022). The executive functions impaired in ADHD have been identified as being those of activation, focus, effort, emotion, memory and action (Brown, 2013). Contemporary conceptualizations of ADHD increasingly apply a strengths-based lens to explore and explain this heterogenous condition (see e.g., Redshaw & McCormack, 2022; Sedgwick et al., 2019). In Aotearoa New Zealand (NZ), Opai (2022) recently contested negative and medicalized conceptualizations of ADHD and offered a Te Ao Māori (Māori world) perspective on the condition (Māori are the indigenous population of NZ). “ADHD has always been seen as a negative term. People could not concentrate, would fiddle, be restless. After much consultation, I turned the essence of the ADHD experience to the positive: aroreretini, (literally, ‘attention goes to many things’)” (Opai, 2022, p. 1182).

Despite these more positive understandings of ADHD, research has consistently identified people with ADHD as experiencing high rates of mental health disorders such as substance use disorders (SUDs), emotional dysregulation, and self-harm. ADHD has also been associated with disruptive, defiant, and antisocial behaviors (Cherkasova et al., 2022; Erskine et al., 2016; Retz et al., 2021; Sayal et al., 2018; Spencer, 2006). People with ADHD often face challenges in interpersonal relationships, education, including suspensions/expulsion from school and lower tertiary enrollment, and employment (Cherkasova et al., 2022; Sayal et al., 2018; Zalsman & Shilton, 2016).

While estimates of ADHD prevalence vary due to methodological differences between studies, it is generally accepted that the global prevalence among children and young people is currently around 5-7% (Freckelton, 2019; Holland et al., 2023; Retz et al., 2021; Sayal et al., 2018; Young & Cocallis, 2022; Zalsman & Shilton, 2016). The male to female ratio of ADHD is estimated to be 3:1 (Slobodin & Davidovitch, 2019; Willcutt, 2012; Young et al., 2020; Zalsman & Shilton, 2016).

A large body of research suggests that ADHD may be a risk factor for interactions with the criminal justice system (CJS). In a meta-analysis of 15,442 children and adolescents (aged 4–15) with ADHD from nine unique samples, ADHD was associated with a two- to threefold increase in the risk of arrests, convictions, and incarcerations in adolescence and adulthood compared to controls without ADHD (Mohr-Jensen & Steinhausen, 2016). Significant associations between ADHD and both convictions and incarcerations were more recently demonstrated in a Danish nationwide longitudinal study, though these effects were smaller than previously reported risks due to adjustment for known individual and familial criminogenic risk factors (Mohr-Jensen et al., 2019). Research has also reported that, compared to individuals without ADHD, those with ADHD are more likely to be younger at first arrest and conviction (Philipp-Wiegmann et al., 2018; Young & Cocallis, 2022) and tend to have an increased risk of recidivism (Retz et al., 2021), and a higher number of further engagements with the CJS (Philipp-Wiegmann et al., 2018).

This increased risk of CJS interactions is also reflected in the disproportionately higher rates of ADHD reported across multiple contact points within the CJS, including police custody/arrests, prison, probation, and forensic mental health settings (Young & Cocallis, 2022). Compared to rates of ADHD in the general population, research suggests that there may be around five times the rate of ADHD in youth offender populations and 10 times the rate of ADHD in adult offender populations (Retz et al., 2021; Young & Cocallis, 2022). In a meta-analysis pooling 102 original studies including 69,997 participants, Baggio et al. (2018) calculated an ADHD prevalence of 26.2% in adult detention populations, corresponding to at least a five-fold over-representation compared to the general population. When retrospectively assessing ADHD rates in childhood, the meta-analytic prevalence of ADHD increased to 41.1% of the sample (Baggio et al., 2018).

Regarding associations between ADHD and offense types, there is a dearth of research available and existing studies have been limited by small convenience samples or cross-sectional designs (Fletcher & Wolfe, 2009), and retrospective, self-report measures of ADHD symptomatology in childhood (e.g., Román-Ithier et al., 2017; Watts, 2018). While, Mohr-Jensen et al. (2019) reported that all recorded offenses (except for murder) were significantly higher for those with ADHD than controls, more research employing large, representative samples is needed to elucidate whether an association exists between ADHD and certain offense types.

Several theories exist on the association between ADHD and CJS interactions. Some suggest that the symptomatology of ADHD may lead to crime due to its associations with low self-control, a widely known criminogenic risk factor (Pratt et al., 2002; van der Maas et al., 2018). Others suggest that impulsive, cognitive, and behavioral symptoms may mean that these individuals are more likely to get caught for crimes committed due to traces left behind (e.g., DNA) (Mohr-Jensen & Steinhausen, 2016). ADHD may also be overrepresented in CJS interactions due to its comorbidity with other psychiatric conditions linked to criminal behaviors, such as conduct disorder, SUDs, and antisocial personality disorder (ASPD) (Pratt et al., 2002; Retz et al., 2021; Sibley et al., 2011). It has also been suggested that impaired socialization and social bonds during development, due to adverse outcomes associated with ADHD symptomatology, may lead to CJS interactions (van der Maas et al., 2018; Watts, 2018). This theorizing is consistent with the social model of disability, which recognizes that people with impairments are disabled by socially constructed barriers including ableist attitudes, systems, structures, and environments (Oliver, 2013; Shakespeare, 2017).

It is therefore important to not only understand how ADHD might lead to interactions with the CJS but also the ways in which ADHD may shape these interactions and affect an individual’s pathway through the justice system. All signatory states to the United Nations Convention on the Rights of Persons with Disabilities (UNCRPD) have a series of legal obligations to protect disabled people (UNCRPD articles 12, 13, 14, and 31). These include ensuring equal recognition before the law and the provision of effective access to justice, protecting the liberty and security of the person (The Office of the High Commissioner for Human Rights, 2006).

However, individuals with ADHD may be at risk from their moment of first contact with the CJS (e.g., police custody and court proceedings) for several reasons. Firstly, they may be perceived as uncooperative or less credible as they are more likely to respond to questions with “don’t know” rather than denying suggestions put to them (possibly due to a lack of confidence in their memory) (Mohr-Jensen & Steinhausen, 2016; Young & Cocallis, 2022). Secondly, individuals with ADHD may be at risk of making false confessions or providing false information, due to the use of inappropriate interviewing techniques (Cunial et al., 2020), greater levels of compliance resulting from difficulties with anxiety or low self-esteem, or due to ADHD symptoms (e.g., restlessness, hyperactivity) motivating individuals to leave the police station (Gudjonsson et al., 2008; Young & Cocallis, 2022).

Signatory states are also required to collect appropriate data to demonstrate they are complying with the obligations set forth in the UNCRPD (Bowden et al., 2022). However, there are considerable challenges in accessing such data. For example, the NZ government has acknowledged a heavy reliance on international prevalence data to inform national response due to current limitations with data collection (Office for Disability Issues, 2019).

Linked population-level health and CJS data from NZ provides an opportunity to fill known gaps in the literature regarding ADHD and CJS interactions. In particular, by enabling analysis of the pathway through the CJS and addressing methodological shortcomings in extant research. They also provide essential data specific to the NZ-context. The aims of the current study are therefore to: (1) Investigate the benefits of using linked administrative data to contribute to research regarding CJS interactions among young people with ADHD; (2) Examine CJS interactions and pathways through the justice system for young adults with ADHD compared to young adults without ADHD; and (3) Assess whether associations exist between ADHD and specific offense types in comparison to individuals without ADHD.

## Methods

### Study Design, Population, and Data

This was a national 3-year birth cohort study using data from Statistics New Zealand’s Integrated Data Infrastructure (IDI), a large database of linked de-identified administrative and survey data about people and households. The IDI is a world-leading resource containing population-level data across a range of life domains including health, education, the labor market, housing, social welfare, and the CJS (see Milne et al. (2019) for a detailed description). The study population consisted of all those born in NZ between 1 July 1992 and 30 June 1995, identified using the Department of Internal Affairs birth records.

**Figure 1.**
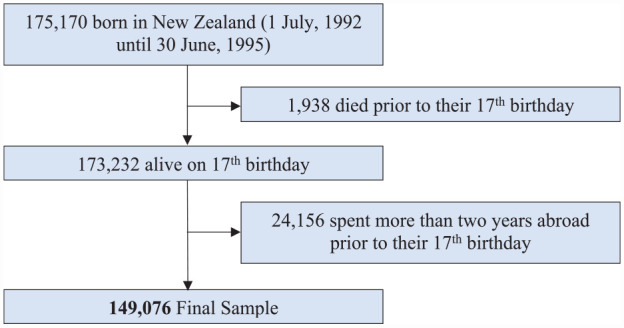
Participant flow chart. *Note*. All counts have been random rounded to base 3.

The observation window for CJS interactions was between the participants’ 17th and 25th birthdays, as New Zealanders are subject to the adult court from age 17 and are considered young adults until 25. This age range is also in keeping with research evidence (Prior et al., 2011). Individuals who spent two or more years outside of NZ or died prior to their 17th birthday were excluded. The final sample consisted of 149,076 individuals (see Figure 1).

### Attention-Deficit/Hyperactivity Disorder

ADHD status was determined using an established case identification method (Bowden, Thabrew, Kokaua, & Braund, 2020; Bowden, Gibb et al., 2020; McLay et al., 2022). This method uses diagnosis codes and inference from medication dispensing contained within four health datasets managed by the NZ Ministry of Health: hospital admissions data from the National Minimum Dataset (NMDS); specialty mental health service use data from the Program for the Integration of Mental Health Data (PRIMHD); subsidized pharmaceutical dispensings from the NZ Pharmaceutical Management Agency (PHARMAC); and the Ministry of Health’s disability support services dataset, Socrates. Codes used to indicate ADHD within each of these datasets are provided in the Supplemental Material (Table S1). Individuals were identified with ADHD if they obtained at least one code relating to an ADHD diagnosis in any of the four datasets. As ADHD is considered to be a lifetime neurodevelopmental condition, case identification reviewed diagnosis data from birth through to the end of the study period.

### Criminal Justice System Measures

Four CJS interaction variables of increasing severity were of interest: police proceedings, court charges, court convictions, and incarcerations. Information on police proceedings was obtained from NZ police recorded offender data. Court charges and convictions were drawn from Ministry of Justice courts data. Incarcerations were obtained from the Department of Corrections dataset. For each CJS interaction variable, individuals were coded as 1 if they had any interaction over an 8-year period from their 17th birthday through to their 25th birthday and 0 for no interaction. Dates for when each type of interaction occurred were also available.

Offense types for criminal convictions were defined using the Australian and New Zealand Standard Offense Classification (ANZSOC), a commonly used method of classifying offense types in Australia and NZ (Australian Bureau of Statistics, 2011). ANZSOC divisions and their descriptions are provided in the Supplemental Material (Table S2). Specific divisions were combined to create different offense types: offenses against people (ANZSOC divisions 1–6); offenses against property (ANZSOC divisions 6–9, and 12); offenses against organizations, government and community (ANZSOC divisions 10–11, and 13–16); and violent offenses (ANZSOC divisions: 2 and 3; ANZSOC subdivisions: murder, attempted murder, abduction and kidnapping, deprivation of liberty/false imprisonment, robbery; and the ANZSOC group manslaughter [does not include driving causing death]). Each offense type was treated as a dichotomous variable (convicted/not convicted). First date of conviction for an offense type was also available.

The seriousness of offenses was defined using the categorization in the Criminal Procedure Act, § 6 (2011). Serious offenses were those punishable by imprisonment of two or more years. This was used to create a dichotomous serious offense indicator. Individuals were coded as 1 if they were convicted of a serious offense, with all other individuals coded as 0. First conviction date for those in the serious offense category was also extracted.

### Sociodemographic Variables

Sociodemographic variables included sex (male/female), age (in years), ethnicity, area of residence, and area-level deprivation. Ethnicity was measured in total response format, allowing individuals to identify with one or more of the following ethnic groups: European; Māori; Pacific; Asian; Middle Eastern, Latin American, African (MELAA); Other. Multiple ethnic identification is a common occurrence in NZ (Statistics New Zealand, 2005). Area-level deprivation and area of residence were based on address information at 17 years of age. Area of residence was categorized into five locations: Auckland; Wellington; rest of the North Island; Canterbury; rest of the South Island. The NZ Deprivation Index 2013 (NZDep2013) was used to capture area-level deprivation. NZDep2013 is based on socioeconomic indicators from the 2013 NZ Census and includes income, employment, home ownership, and educational attainment. The index is used to assign decile values (ranging from 1 to 10) to the meshblocks (census areas) that individuals live in. In the current study, decile values were collapsed to quintiles with quintile 1 indicating areas of the least deprivation and quintile 5 indicating the greatest deprivation.

### Data Analysis

The sociodemographic characteristics of the participant population were described descriptively, stratified by ADHD status. Observed rates of each of the four CJS interaction types and conviction offense types were calculated for those with and without ADHD. Cox proportional hazards models were employed to examine associations between ADHD and each CJS interaction (proceedings, charges, convictions, and incarcerations). Unadjusted and adjusted hazard ratios (HRs) for ADHD with 95% confidence intervals (CIs) were estimated using the robust variance estimator (Lin & Wei, 1989). Adjusted analyses included sex, age, ethnicity, area-level deprivation, and area of residence in the model. Participants were right censored if they died, traveled overseas for a period greater than 3 consecutive months (92 days) or reached the end of the study period (i.e., their 25th birthday) without experiencing the relevant CJS interaction. Associations between ADHD and conviction for different offense types were also examined, replicating the cox proportional hazards analysis described. The decision was made to focus on convictions, as this is the first interaction type in the system that appears on an individual’s criminal record.

Data management was undertaken in SAS Enterprise Guide version 7.1 (SAS Institute Inc, 2014) and analyses were conducted in Stata MP version 15 (StataCorp, 2017). As per the confidentiality requirements of Statistics New Zealand, raw counts were suppressed if below 20 for CJS interactions and below 6 for all other variables. All counts were randomly rounded to base 3.

## Results

### Participant Population

Of the 149,076 individuals in our final sample, 2.7% (*n* = 3,975) were identified as having ADHD. The sociodemographic characteristics for those with and without ADHD in our sample are presented in Table 1. Those with ADHD were more likely to be male and identify as European, relative to those without ADHD. In contrast, there were fewer Māori, Pasifika, and Asian individuals amongst those with ADHD relative to those without. Across both ADHD and non-ADHD groups, there was a similar proportion of individuals in each deprivation quintile, with a slightly greater proportion of people without ADHD in the most deprived quintile. The vast majority of participants lived in the North Island of NZ (Auckland, Wellington and Rest of North Island), 73.1% of those with ADHD and 75.8% of those without ADHD. The ADHD group had fewer individuals residing in Auckland and the rest of the North Island, relative to those without ADHD.

**Table 1.** Sociodemographic Characteristics of Those With and Without ADHD at Baseline (age 17).

	ADHD *n* (%)	Without ADHD *n* (%)
Sex		
Male	3,057 (76.9%)	73,680 (50.8%)
Female	918 (23.1%)	71,418 (49.2%)
Ethnicity		
European	3,573 (89.9%)	111,126 (76.6%)
Māori	1,017 (25.6%)	43,125 (29.7%)
Pasifika	174 (4.4%)	16,680 (11.5%)
Asian	84 (2.1%)	6,804 (4.7%)
MELAA	42 (1.1%)	1,392 (1.0%)
Other	21 (0.5%)	1,095 (0.8%)
Socioeconomic Deprivation		
Quintile 1 (least deprived)	747 (18.8%)	28,326 (19.5%)
Quintile 2	720 (18.1%)	26,343 (18.2%)
Quintile 3	783 (19.7%)	25,764 (17.8%)
Quintile 4	828 (20.8%)	26,931 (18.6%)
Quintile 5 (most deprived)	876 (22.0%)	34,554 (23.8%)
Missing	24 (0.6%)	3,183 (2.2%)
Region		
Auckland	1,020 (25.7%)	41,004 (28.3%)
Wellington	510 (12.8%)	15,084 (10.4%)
Rest of N. Island	1,377 (34.6%)	53,892 (37.1%)
Canterbury	567 (14.3%)	17,415 (12.0%)
Rest of S. Island	498 (12.5%)	16,128 (11.1%)
Missing	S	1,578 (1.1%)

*Note*. All counts have been random rounded to base 3. S indicates suppression due to small counts (<6). MELAA = Middle Eastern, Latin American, African.

### Criminal Justice System Interactions

**Figure 2.**
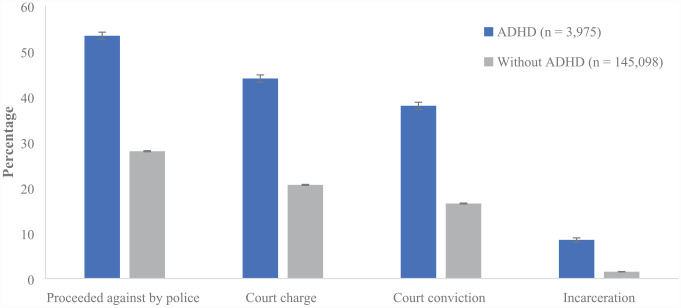
CJS interactions by ADHD status. *Note*. CJS = Criminal Justice System.

Observed rates indicate that all CJS interactions were more common in those with ADHD than those without (Figure 2). In those with ADHD, a considerable proportion, 53.4%, were proceeded against by police by the age of 25 years. Court charges were laid against 44.0%, 38.0% had received a court conviction, and 8.5% were incarcerated. Comparatively, 28.0% of those without ADHD were proceeded against by police, 20.6% were charged in court, 16.5% received a conviction, and 1.5% were incarcerated.

Cox proportion hazards analyses indicated that those with ADHD were significantly more likely to interact with the CJS relative to those without ADHD (Table 2). Moreover, the likelihood of CJS interactions for those with ADHD increased through the CJS pathway from an adjusted HR of 2.1 for police proceedings, 2.2 for court charges, 2.3 for court convictions, and a substantial increase to 4.8 for incarceration.

**Table 2.** Summary of Hazard Ratios Associated With ADHD for Each Level of CJS Interactions.

	*n*	Unadjusted HR (95% CI)	Adjusted^a^ HR (95% CI)
Overall			
Proceeded against by police	2,121	2.34 [2.24, 2.45]	2.07 [1.98, 2.17]
Court charge	1,749	2.50 [2.38, 2.62]	2.21 [2.10, 2.32]
Court conviction	1,512	2.62 [2.48, 2.76]	2.31 [2.19, 2.43]
Incarceration	339	5.51 [4.92, 6.18]	4.81 [4.28, 5.41]

*Note*. All counts have been random rounded to base 3. CJS = Criminal Justice System; HR = Hazard ratio; CI = Confidence interval.

a Adjusted for gender, ethnicity, deprivation, and area of residence.

### Offenses Types

Observed rates for conviction offense type by ADHD status and unadjusted and adjusted HRs are presented in Table 3. Individuals with ADHD had higher observed rates for all offense types relative to those without ADHD. Likewise, cox proportional hazard analyses indicated that ADHD was associated with significantly increased likelihood of all offense types relative to those without ADHD. The adjusted hazards of conviction for serious offenses (HR 3.5), violent offenses (HR 3.7), and offenses against property (HR 3.8) were particularly high.

**Table 3.** Summary Statistics and Hazard Ratios Associated With ADHD Status for Offense Types.

	ADHD (*N* = 3,975) *n* %	Non-ADHD (*N* = 145,098) *n* %	Unadjusted HR (95% CI)	Adjusted^a^ HR (95% CI)
Serious offenses	882 (22.2%)	8,541 (5.9%)	4.02 [3.74, 4.33]	3.51 [3.26, 3.78]
Offenses against the person	999 (25.1%)	11,256 (7.8%)	3.47 [3.25, 3.70]	2.79 [2.62, 2.98]
Violent offenses	627 (15.8%)	5,847 (4.0%)	4.16 [3.83, 4.52]	3.65 [3.35, 3.97]
Offenses against property	861 (21.6%)	8,094 (5.6%)	4.16 [3.88, 4.47]	3.78 [3.52, 4.06]
Offenses against organizations, government and community	1,296 (32.6%)	20,463 (14.1%)	2.57 [2.43, 2.72]	2.24 [2.12, 2.37]

*Note*. All counts have been random rounded to base 3. HR = Hazard ratio; CI = Confidence interval.

a Adjusted for gender, ethnicity, deprivation, and area of residence.

## Discussion

The current study utilized linked population-level health and CJS data to examine interactions with, and pathways through, the CJS for young adults with and without ADHD in NZ. Findings revealed that over half of young adults with ADHD interacted with the CJS by their 25th birthday. Moreover, when controlling for important sociodemographic factors, individuals with ADHD were over two times as likely to be proceeded against by police and charged or convicted in court and almost five times as likely to be incarcerated than those without ADHD. Analyses also revealed that for all offense types, those with ADHD were between two to almost four times as likely to be convicted compared to those without ADHD.

Our finding of an increased risk of being proceeded against by police, being charged or convicted in court, and being incarcerated amongst those with ADHD is consistent with prior research in this area (Erskine et al., 2016; Mohr-Jensen & Steinhausen, 2016; Mohr-Jensen et al., 2019). However, in contrast to previous studies, we also observed a clear pattern of increasing risk of CJS interactions as individuals with ADHD proceeded through the system. More specifically, while the effect sizes we observed for the risk of police proceedings, court charges and court convictions fall within the range of those previously reported for people with ADHD, for incarcerations we report a substantially higher risk (Mohr-Jensen & Steinhausen, 2016; Mohr-Jensen et al., 2019). The greater effect size for incarceration observed in our study may be due to the lack of control for comorbid conditions such as CD, which are known criminogenic risk factors (Pratt et al., 2002), or the possibility of the case identification method used in the current study capturing more severe cases of ADHD (Bowden, Gibb et al., 2020). The sharp increase in the risk of incarceration observed may also signal differences in the NZ justice system’s approach to ADHD, which may be less responsive to the condition than other nations, particularly the steps in the justice system between conviction and sentence. This would suggest that the UNCRPD obligations of equal recognition before the law and the elimination of discrimination on the basis of disability are not being met for individuals with ADHD in NZ.

The finding indicating that ADHD is associated with an increased risk across the spectrum of conviction offense types is consistent with the limited research to date (Retz et al., 2021). Our findings that the largest effect sizes were observed for serious offenses, violent offenses, and offenses against property broadly reflect those of Mohr-Jensen et al. (2019). These findings may support the idea of an association between ADHD and more impulsive crimes (e.g., burglary and theft), particularly for individuals with higher levels of impulsivity (Fletcher & Wolfe, 2009). Symptoms of impulsivity may also explain associations between ADHD and violent crimes such as assault but only when these crimes occur in a reactive and not proactive/premeditated context (Retz & Rösler, 2009). Retz and Rösler (2009) suggest that a relationship between ADHD and reactive aggression may be feasible due to the psychopathological characteristics of ADHD. Reactive aggression tends to be driven by affective outbursts and impulsivity. It is not premeditated but arises spontaneously, generally as a reaction to conflict or provocation with the sole aim of reducing tension and agitation (Retz & Rösler, 2009). This may explain a link between violent crime at least in a reactive context and the hyperactive-impulsive symptoms of ADHD (Retz & Rösler, 2009).

### Implications

The overrepresentation of individuals with ADHD at each stage of the CJS suggests that the NZ CJS is failing to identify individuals with ADHD either before or during their interactions with the legal system, or that the legal system is correctly identifying these individuals but failing to respond to and make appropriate accommodations. The identification and consideration of ADHD is important because due to the symptoms of the condition, these individuals may be at risk from their moment of first contact with the CJS. Moreover, it has been suggested that individuals with ADHD may find the nature of incarceration more difficult than those without the condition (Baggio et al., 2018; Freckelton, 2019). Compared to offenders without ADHD, individuals with ADHD have been shown to be more likely to engage in misconduct in prison such as verbal or physical aggression, damage to property, and incidents involving self-injury (e.g., self-harm or suicidal behaviors) (Baggio et al., 2018; Retz et al., 2021; Young & Cocallis, 2022). This may be due to symptoms such as hyperactivity or impulsivity impairing an individual’s ability to effectively cope with the structure and demands of imprisonment which may lead to disciplinary infractions, the prevention of early release, and even further convictions and extended sentences (Young & Cocallis, 2022). As such, it has been suggested that punishment via incarceration may not be effective in preventing recidivism in those with ADHD (Mohr-Jensen et al., 2019). It has also been suggested that during sentencing the potential for incarceration to exacerbate symptoms, increase the risk of mistreatment, or be a more difficult experience than for people without ADHD may need to be considered (Freckelton, 2019). The failure of any CJS to screen for or respond appropriately to ADHD may therefore have serious implications such as causing a subjectively more painful and less productive experience for a reasonable proportion of offenders (Pratt et al., 2002). A recent NZ justice system initiative has emerged in recognition of the need to respond more quickly and more effectively to neurodivergence (Walker & Doogue, 2019). At this stage, there is no mechanism within the initiative to screen for specific neurodiverse conditions, including ADHD (Clasby et al., 2022).

The current study also provides NZ data on ADHD in the CJS for the first time and demonstrates an ability to use linked administrative data to help NZ meet its UNCRPD obligation of collecting disability data (Article 31). Such methodology could be periodically replicated and expanded upon both in NZ and abroad for better monitoring of States Parties progress toward meeting their obligations under the UNCPRD in relation to the CJS. In particular, the use of integrated data to enable analysis of the pathway through the CJS at a population level is novel and provides more nuanced insights into the disabling experiences of young adults with ADHD in the CJS.

### Strengths and Limitations

The current study has a number of strengths. Firstly, we were able to establish a large, 3-year national birth cohort, tracking participants until their 25th birthday. The use of linked data allowed us to identify those with and without ADHD and examine their interactions with the CJS at multiple contact points including police, courts, and corrections. We were also able to explore differences in the types of offending for those with compared to those without ADHD, which allowed us to investigate how their pathways might differ from one another. Additionally, the use of linked data meant that we were able to control for various sociodemographic factors and account for early exits during the observation period (e.g., emigration and death).

There are several limitations to the current study that should be noted. We did not control for the effects of conditions highly comorbid with ADHD such as CD and SUDs, which may be associated with an increased risk of delinquency and crime (Pratt et al., 2002; Retz et al., 2021; Sibley et al., 2011). While some studies have shown an independent effect of ADHD on offending when controlling for comorbid conditions (e.g., Sibley et al., 2011), other research has shown no independent effect of ADHD on crime when controlling for comorbidities (e.g., Mordre et al., 2011; Retz et al., 2021). This was considered out of scope for the present study in part because the data source used (the IDI) is not effective in capturing co-occurring conditions of interest such as CD (Bowden, Gibb et al., 2020). However, we note that by focusing on what we know we can capture in the IDI, it allows us to consider whether approaches can be developed based on a screening for ADHD, regardless of the presence of co-occurring conditions.

The use of administrative data for identifying ADHD likely undercounts, and may falsely identify, cases. For example, the method relies on health service use and will not capture individuals with ADHD who are not accessing treatments (Milne et al., 2022). Alternatively, the use of pharmaceuticals as indications of ADHD may falsely capture individuals who may be accessing these medications for other conditions (D’Souza et al., 2020). The level of undercount is reflected in the relatively low prevalence of ADHD found in our sample at 2.7% compared with worldwide prevalence estimates typically in the 5-7% range (Freckelton, 2019; Holland et al., 2023; Retz et al., 2021; Young & Cocallis, 2022). The effect that any misclassification bias may have had on our findings is unknown. As our case identification method may also capture more complex or severe cases of ADHD, this may partly explain the increasing risk of CJS interactions observed as individuals with ADHD moved through the justice system. Lastly, as our cohort excludes migrants to NZ who make up almost one-third of young people between 20 and 24 years of age living in the country (Statistics New Zealand, 2013), it is only representative of those born in NZ and not the population as a whole.

### Further Research

To fully elucidate and understand the relationship between ADHD and CJS interactions, there is a need for more research employing large, representative samples with longitudinal rather than cross-sectional designs. The importance of controlling for co-occurring psychiatric conditions such as CD and SUDs has been highlighted by a number of previous studies, due to their independent associations with crime, and the extent to which this impacts CJS interactions among individuals with ADHD in NZ needs to be determined. Future research could also benefit from exploring the role of other risk and protective factors for CJS interactions among those with ADHD in NZ. For example, research has noted that while co-occurring autism may be a protective factor against crime in those with ADHD (Mohr-Jensen et al., 2019), adverse outcomes across schooling and employment that are associated with ADHD may interact and exacerbate one another, increasing the likelihood of delinquency (Erskine et al., 2016). The potential benefits of early intervention and treatment of ADHD have also been highlighted with evidence that active periods of treatment with ADHD medication may be associated with reductions in criminality (Philipp-Wiegmann et al., 2018; Retz et al., 2021) and a significant risk reduction for convictions and incarcerations (Mohr-Jensen et al., 2019). This may also disrupt any pathways that may exist from ADHD to the development of SUDs. It has been suggested that multimodal interventions may be most effective, with medication reducing the symptoms of ADHD to increase engagement with the non-pharmacological side of treatment (e.g., CBT) (Retz et al., 2021). More research is needed to examine the efficacy of early intervention and treatment for ADHD on CJS outcomes in a NZ context. It would also assist policy makers to understand how various participants in the CJS become aware of information on ADHD and whether legal processes and procedures are altered in response to such information.

Further research is necessary to understand why the risk of CJS interactions increases so sharply from conviction to sentence for young persons with ADHD. A better understanding of how young persons with ADHD experience that pathway will inform how best to mitigate this disabling experience. This could present a significant opportunity for NZ and other countries to innovate, including by allowing for supports, accommodation, and alternative pathways (e.g., restorative justice) in order to ensure obligations under the UNCRPD are met.

## Conclusion

Our findings revealed that not only were individuals with ADHD overrepresented at all stages of the CJS and offense types examined, there was also a pattern of increasing risk for CJS interactions as these individuals moved through the system. These results highlight the importance of early identification and responsivity to ADHD within the CJS and suggest that the NZ justice system may require changes to both of these areas to ensure that young individuals with ADHD receive equitable access to, and treatment within, the CJS. The current study has also revealed the utility of effective data linkage in better understanding the pathways of individuals with ADHD through the CJS.

## Supplemental Material

sj-docx-1-jad-10.1177_10870547231177469 – Supplemental material for Risk of Criminal Justice System Interactions in Young Adults with Attention-Deficit/Hyperactivity Disorder: Findings From a National Birth Cohort Click here for additional data file. Supplemental material, sj-docx-1-jad-10.1177_10870547231177469 for Risk of Criminal Justice System Interactions in Young Adults with Attention-Deficit/Hyperactivity Disorder: Findings From a National Birth Cohort by Francesca Anns, Stephanie D�Souza, Conrad MacCormick, Brigit Mirfin-Veitch, Betony Clasby, Nathan Hughes, Warren Forster, Eden Tuisaula and Nicholas Bowden in Journal of Attention Disorders
