# Supplementary material for: Risk of Criminal Justice System Interactions in Young Adults with Attention-Deficit/Hyperactivity Disorder: Findings From a National Birth Cohort
Source: J Atten Disord. 2023 May 30;27(12):1332–42. doi: 10.1177/10870547231177469 (PMC10498656; doi:10.1177/10870547231177469)
Supplement: sj-docx-1-jad-10.1177_10870547231177469 – Supplemental material for Risk of Criminal Justice System Interactions in Young Adults with Attention-Deficit/Hyperactivity Disorder: Findings From a National Birth Cohort [file sj-docx-1-jad-10.1177_10870547231177469.docx]

**Supplementary Material**

| **Table S1.**  **ADHD ascertainment from NMDS, PRIMHD, PHARMAC, and Socrates data** | | | | |
| --- | --- | --- | --- | --- |
| **ICD-10-AM codes** | **ICD-9-CMA II** | **DSM-IV codes** | **PHARMAC chemical IDs (description)** | **Socrates assigned diagnosis codes** |
| F90.0, F90.8, F90.9 | 314.00,  314.01 | 314.00, 314.01, 314.9 | 1389 (dexamfetamine sulfate), 1809 (methylphenidate hydrochloride), 3880 (methylphenidate hydrochloride extended-release), 3887 (atomoxetine) | 1201 |
| *Note:* Codes were based on ADHD case identification in Bowden et al. (2020); ICD-10-AM codes were used for ADHD ascertainment within NMDS and PRIMHD datasets. ICD-9-CMA II codes were used for records prior to July 1999; DSM-IV codes were used for ADHD ascertainment within the PRIMHD dataset. | | | | |

| **Table S2.**  **ANZSOC Divisions** | |
| --- | --- |
| **Division** | **Description** |
| 1 | Homicide and related offences |
| 2 | Acts intended to cause injury |
| 3 | Sexual assault and related offences |
| 4 | Dangerous or negligent acts endangering persons |
| 5 | Abduction, harassment and other offences against the person |
| 6 | Robbery, extortion and related offences |
| 7 | Unlawful entry with intent/burglary, break and enter |
| 8 | Theft and related offences |
| 9 | Fraud, deception and related offences |
| 10 | Illicit drug offences |
| 11 | Prohibited and regulated weapons and explosives offences |
| 12 | Property damage and environmental pollution |
| 13 | Public order offences |
| 14 | Traffic and vehicle regulatory offences |
| 15 | Offences against government procedures, government security and government operations |
| 16 | Miscellaneous offences |
